# Supplementary material for: Dissociable electrophysiological correlates of semantic access of motor and non-motor concepts
Source: Sci Rep. 2019 Aug 7;9:11482. doi: 10.1038/s41598-019-47835-9 (PMC6686022; doi:10.1038/s41598-019-47835-9)
Supplement: Supplementary file 1 — Related Manuscript File [file 41598_2019_47835_MOESM1_ESM.pdf]

Dissociable electrophysiological correlates of semantic access of motor and non-motor concepts

Authors:

Rodika Sokoliuk* (r.sokoliuk@bham.ac.uk; corresponding author)[1,3]

Sara Calzolari (s.calzolari@bham.ac.uk)[1,2,3]

Damian Cruse (d.cruse@bham.ac.uk)[1,3]

1. School of Psychology, University of Birmingham, Birmingham, England, B15 2TT
2. Department of General Psychology, University of Padova, Padova, Italy
3. Centre for Human Brain Health, University of Birmingham, Birmingham, England, B15 2TT

Supplementary material

Complete list of stimuli:

<u>MOTOR VERBS</u>:

catch - skip - flick - rub - stir - scrub - push - grasp - pull - seize - chew - prod - smack - drag - shrug - squeeze - flex - throw - slam - snip - speak - pinch - bend - slap - hurl - hold - carve - reach - give - hit - grab - dab - stretch

<u>NON-MOTOR VERBS</u>:

fail - pray - burn - grow - melt - shrink - wed - blush - teach - beg – praise- shine - plead - taint - cheat - faint - spoil - starve - count - fade - thaw - learn - boil - sweat - warn - scare - wilt - win - tease - join - swear - quit – tempt

Validation of source analysis of early ERP components:

<u>Methods</u>:

We performed a validation of our source estimation method by localizing brain activity of the P1 and N1 components (across all trials of both conditions) in the time windows 106-160ms (P1) and 164-203ms (N1) respectively. We obtained the latencies for these time windows by computing the time course of the average VEP over occipital electrodes and identifying peaks within the classic P1 and N1 latency windows (cf. Figure S1 in yellow and orange).

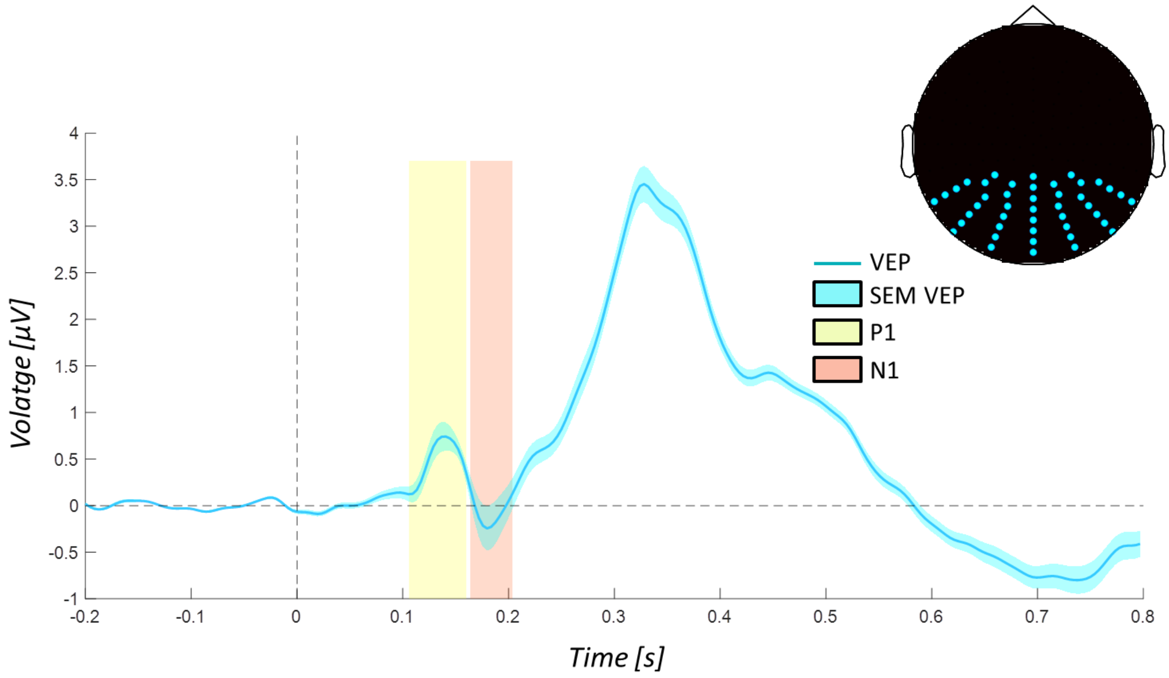

Figure S1: Defining time windows of interest for validation. *Time windows of interest were defined by computing the average Visual Evoked Potential (VEP; here in light blue, cyan shaded area depicts standard error of the mean) over*

occipital electrodes (depicted in light blue in topography plot) and identifying time windows around latencies for P1 (yellow) and N1 (orange).

To source localise these components, we followed the LCMV ERP beamforming method described recently by members of the Fieldtrip group [1]. Specifically, first, the data were filtered between 1 and 40Hz using a firws filter with default parameters of fieldtrip's "ft_preprocessing" function. Then, the sensor co-variance matrix of that data was estimated over a time window, containing pre- and post-stimulus period (-500 - +500ms relative to stimulus presentation) and a spatial filter was computed over this time window. The parameters used here were taken from Popov et al., 2018 and involved applying a fixed dipole orientation, weights normalization (to reduce the centre of the head bias), and setting the regularisation parameter lambda to 5% to increase the signal to noise ratio. Time courses of source estimates of poststimulus time windows were then extracted as the saved dipole moments (.mom fields in fieldtrip) and the average over the absolute values in that time window was computed for every grid point.

As can be seen in Figure S2 below, this approach led to appropriate source estimates with occipital clusters for the P1 and N1 time windows and was thus used for the source analysis of the ERP effect we found on the sensor level.

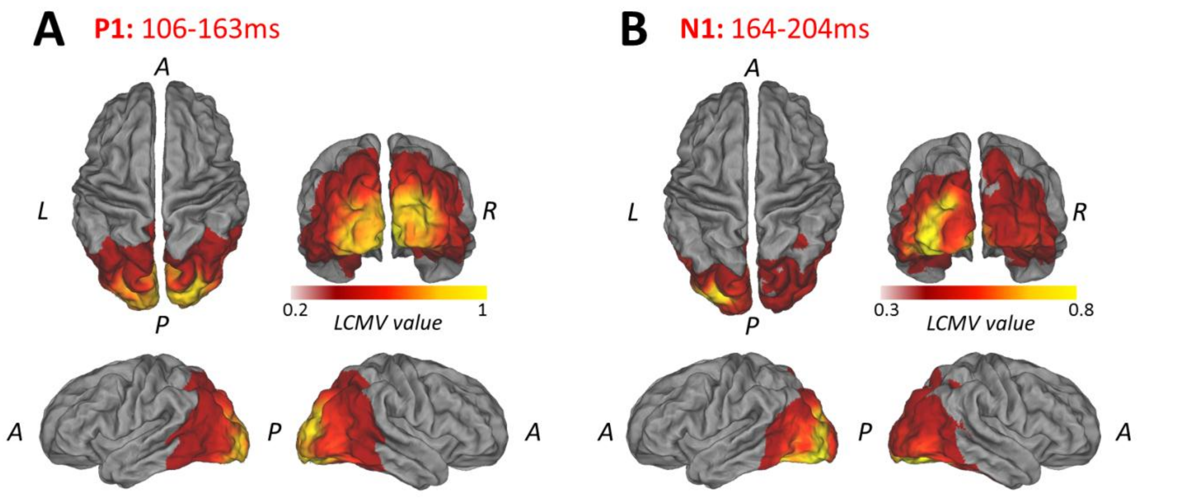

Figure S2: Source estimate of early VEP components. (A) *Source estimate of P1 component, averaged over time window 106-163ms.* ***(B)*** *Source estimate of N1 components, averaged over time window 164-203ms. (Abbreviations indicate anatomical landmarks: L=left; R=right; A=anterior; P=posterior; results interpolated onto MNI brain and thresholded).*

References

1. Popov, T., Oostenveld, R. & Schoffelen, J. M. FieldTrip made easy: An analysis protocol for group analysis of the auditory steady state brain response in time, frequency, and space. *Front. Neurosci.* **12,** 1–11 (2018).
